# Supplementary material for: SPAligner: alignment of long diverged molecular sequences to assembly graphs
Source: BMC Bioinformatics. 2020 Jul 24;21(Suppl 12):306. doi: 10.1186/s12859-020-03590-7 (PMC7379835; doi:10.1186/s12859-020-03590-7)
Supplement: Supplementary file 1 — Additional file 1 Supplementary file contains additional algorithms and datasets description (bmc_supplement.pdf). [file 12859_2020_3590_MOESM1_ESM.pdf]

Supplementary Material for "SPAligner: alignment of long diverged molecular sequences to assembly graphs"

1 Alignment extension heuristics and thresholds

In this section we list the heuristics and constraints used by SPAligner during the alignment extension process (e.g. construction and processing of the alignment graphs). Despite significantly improving the overall running-time, some of them may also result in recovery of sub-optimal alignment paths. The default parameter values were set to maintain reasonable speed/accuracy trade-offs.

- While constructing an alignment graph $SG(G, S)$ for finding an optimal alignment path of nucleotide sequence S between positions s and e in graph G, only the subgraph of G representing the union of paths shorter than $\alpha \cdot |S|$ ($\alpha = 1.3$ by default) between s and e is considered.
- Search for the optimal filling path between consecutive anchors in the alignment skeleton (see section "Sequence to graph alignment via alignment graphs") is aborted if number of vertices in the corresponding alignment graph exceeds max_gs_states threshold (by default equal to $120 \cdot 10^6$).
- Maximal weight of the optimal path in the alignment graph $SG(G, S)$ is limited by a fraction of the length of nucleotide sequence $|S|$ (by default $|S|/5$).
- While searching for an optimal alignment path of nucleotide sequence S between positions s and e in graph G a straightforward enumeration of a limited number of paths between the two positions (by default 5000) is performed. Edlib library [1] is used to find the alignment scores of their sequences against S. Minimal attained score is then used to further bound the weight of the optimal path.
- While finding the optimal alignment paths of the nucleotide sequence fragment Suf beyond rightmost anchor, the minimal alignment score $min\_score(i)$ is maintained for every prefix $Suf[0 : i]$. At any moment, the score for any alignment graph vertex, corresponding to the prefix $Suf[0 : i]$ is additionally bounded by $min\_score(i) + i \cdot penalty\_ratio$ ($penalty\_ratio = 0.1$ by default). To further prevent potential performance issues, the search is performed only if $|Suf|$ does not exceed a certain threshold (5 Kb by default).
- Upper bounds are introduced on the number of entries within the priority queue within Dijkstra algorithm as well as the total number of queue extraction events (default value is 10^6 for both). Whenever any of the limits is exceeded, the search is aborted.
- While aligning amino acid sequences, the alignment path can not be extended beyond a stop codon.

2 Support for split-read alignments

SPAligner was primarily developed to find semi-global sequence alignments. But if the path satisfying the constraints for the entire query can not be identified, the procedure might result in several (query-disjoint) alignments. For example, whenever the search for an appropriate path between two consecutive skeleton anchors fails (see Section "Alignment of long nucleotide sequences"), the alignment is divided into two parts.

Moreover, in the general case, "anchor chaining" step is repeated several times for a single query. After the skeleton chain is identified anchors with query ranges spanned by the chain are discarded. The process is repeated while there are anchor alignments to consider.

"Reconstruction of the filling paths" is then invoked independently for each resulting chain.

3 Shortest paths search in binary-weighted graphs

Straightforward approach of aligning query S to graph G (both with fixed or arbitrary start/end positions) via searching the appropriate minimal-weight path in alignment graph with Dijkstra algorithm (see section "Sequence to graph alignment via alignment graphs"; [2, 3]) has the worst-case time complexity of $O(|G| \cdot |S| \cdot \log(|G| \cdot |S|))$. Several improved algorithms [4, 5] with the time complexity of $O(|G| \cdot |Sub|)$ have been earlier suggested for an important case of μ and σ equal to 1 (edit distance alignment cost).

We note that the same time complexity can be achieved by a slight modification of Dijkstra algorithm.

The edges of $SG(G, Sub)$ under the edit distance score have weights of either 0 or 1. It is easy to see that at any moment of searching for the shortest path in such binary-weighted graph, the priority queue within Dijkstra algorithm contains elements with no more than two distinct priority values, which can only differ by 1. Thus the priority queue can be replaced by a deque: all the vertices across 0-edges (1-edges) are appended along with the corresponding distance to the beginning (to the end) of deque. Deque implementation provides all necessary operations in $O(1)$ time, improving the overall running time to $O(|G| \cdot |S|)$.

4 Leveraging fast sequence-to-sequence alignment methods

We implemented a modification of the approach described in Section "Sequence to graph alignment via alignment graphs", which in practice benefits from available highly optimized solutions for sequence alignment. We define a new graph $SG(G, Sub)$ over a subset of vertices of $SG(G, Sub)$. For two particular anchors a and b $SG(G, Sub)$ have vertices corresponding to

- $< p_a, 0 >$, where $p_a = (e(a), end_e(a))$,
- $< p_b, |Sub| >$, where $p_b = (e(b), start_e(b))$,
- $< p_{start}(e), pos_{Sub} >$ and $< p_{end}(e), pos_{Sub} >$, where $p_{start}(e) = (e, 0)$, $p_{end}(e) = (e, |e|)$ and e iterates through all edges of G and $pos_{Sub} \in [0, |Sub|]$.

Edges of $SG(G, Sub)$ are defined as follows:

- $< p_{start}(e), pos_{Sub} > \to < p_{end}(e), pos_{Sub} + c >$ of length $ED(e, Sub[pos_{Sub} : pos_{Sub} + c])$,
- $< p_a, 0 > \to < p_end(e(a)), c >$ of length $ED(e(a)[end_e(a) : |e(a)|], Sub[0 : c])$,
- $< p_start(e(b)), pos_Sub > \to < p_b, |Sub| >$ of length $ED(e(b)[0 : start_e(b)], Sub[pos_{Sub} : |Sub|]$,
- $< p_end, pos_Sub > \to < p_start, pos_Sub >$ of weight zero if p_{end} and p_{start} both corresponds to the same vertex in G (i.e. $p_{end} = (e_1, |e_1|)$, $p_{start} = (e_2, 0)$ and the end of edge e_1 is same vertex as the start of e_2),

where e iterates through all edges of G and Sub_{pos} and c – through all permissible positions in Sub.

While searching for the shortest path in $SG(G, Sub)$, with Dijkstra algorithm, SPAligner calculates distances from vertex $< p_{start}(e)$, $pos_{Sub} >$ to vertices $< p_{end}(e)$, $pos_{Sub} + c >$ for all values of c in a single call to the Edlib library [1]. Due to restrictions of Edlib library this implementation only supports edit distance (μ and σ are equal to 1). SPAligner further enhance the optimal alignment finding approach presented above by various heuristics listed in Supplement Section "Alignment extension heuristics and thresholds".

5 Generation of simulated reads and assembly graphs

Reference and dataset availability information is summarized in Table S1.
Assembly graphs were generated by SPAdes-3.12.0 [6] assembler with parameter -k 21,33,55,77.

Simulated PacBio reads were generated by Pbsim [7] with option −−model_qc data/model_qc_clr. Simulated Nanopore reads were generated by NanoSim [8] with default parameters and option circular for *E. coli* and linear for *C. elegans* and *S. cerevisiae.* While by default Nanosim generates a set of simulated reads which consists of "perfect" reads (sequences directly from genome), "unaligned" reads (with error rate over 90%) and "aligned" reads (with the same error rate as training reads), in our experiments we used only "aligned" reads, which are closer to real data. Simulated Illumina reads for *C. elegans* were generated by ART-MountRainier-2016-06-05 [9].

Species	Reference	Illumina reads	PacBio reads	Nanopore reads
E. coli K12	U00096.2	ERA000206	PacificBiosciences DevNet	Loman Lab R9 data
S. cerevisiae S288C	GCA_000146045.2	ERP016443	ERR1655118	ERP016443
C. elegans Bristol N2	GCA_000002985.3	–	PacificBiosciences DevNet	PRJEB22098

Supplementary Table S 1: Due to lack of freely available up-to-date ONT data for strain Bristol N2, reads sequenced from closely-related wild-type *C. elegans* strain VC2010 [10] were used (see original study for discussion of their relatedness).

6 Notes on running aligners

Full archive with benchmarking data and scripts can be uploaded from https://figshare.com/s/b0dc3f715e0224e3e962

SPAligner. SPAligner accepts query sequences in fastq/fasta format (gzip archives supported) and assembly graphs in GFA1 format (gfa-spec.github.io/GFA-spec) from widely used short-read assemblers (e.g. SPAdes [6], Minia [11], Megahit [12]) and tools for de Bruijn graph construction (e.g. BCALM2 [13] or gbuilder from SPAdes distribution).

In general, SPAligner imposes the following major restriction on the graph structure: The overlap sizes between segments in GFA file (given by "L" records) must be explicitly provided and have the same value. Moreover, current implementation requires overlap sizes to have an odd value. Note that while providing an integer K as a parameter for Minia and BCALM2, the resulting size of the overlaps is $K - 1$, whereas for SPAdes and Megahit it is K.

Resulting alignments are output in custom tsv and fasta formats. In our benchmarks SPAligner was run with default settings.

vg. Assembly graphs were first converted to vg format by the "vg mod -X 1024" command. For long read alignment we used the appropriate mode of the "vg map" command ("vg map -m long"), leaving other parameters to defaults. For each query sequence vg provides a list of non-overlapping local alignments ordered by their position on a read. Resulting local alignments were further processed to infer the longest continuous path P consisting of edges $e_1, \ldots, e_n$ with consecutive local alignments (P starts from the beginning of the first local alignment on e_1 and ends on the final position of the last local alignment on e_n).

GraphAligner. GraphAligner accepts assembly graphs in GFA format and outputs alignment in custom json format. GraphAligner was run with default settings. For *E. coli* datasets we additionally ran it with "try-all-seeds" flag recommended by the developers for bacterial data.

7 Supplementary Table S2

Aligner	MR(%)	AI (%)	T (h:m)	M (Gb)	MR(%)	AI (%)	T (h:m)	M (Gb)
E. coli PacBio					*E. coli* ONT			
vg	9	83	00:16	3.9	68	87	01:12	4.2
GraphAligner	99.8	82.2	00:01	0.1	96.1	86.8	00:01	0.1
GA try-all-seeds	99.8	82.2	00:01	0.1	96.2	86.8	01:57	7
SPAligner	99.8	82.2	00:01	0.2	96.5	86.6	00:02	0.4
S. cerevisiae PacBio					*S. cerevisiae* ONT			
vg	8	83	07:55	90	21	83	14:13	92
GraphAligner	99	82.35	00:01	0.2	85	82.38	00:01	0.2
SPAligner	98.8	82.31	00:10	0.6	85.6	82.1	00:20	0.7
C. elegans PacBio					*C. elegans* ONT			
vg	7	83	27:51	314	20	88	87:26	301
GraphAligner	99.2	82.6	00:01	1.3	80.1	87.3	00:03	1.7
SPAligner	98.4	82.5	0:16	1.1	88.8	86.8	00:33	1.5

Supplementary Table S 2: Summary statistics of aligning simulated PacBio/ONT reads to short-read assembly graphs (constructed by SPAdes with k-mer size equal to 77). Each dataset consists of 10k reads longer than 2 Kbp. All runs performed in 16 threads. While the number of mapped reads increases by 10-15% (as compared to real datasets) across all tools, similar conclusions about tools efficiency can be made. Specifically, SPAligner showed the best performance with respect to the number of mapped reads (for ONT datasets) and memory, GraphAligner showed the best performance with respect to speed, and SPAligner and GraphAligner showed roughly the same performance with respect to average identity.

8 Supplementary Table S3

	E. coli PacBio(%)	*E. coli* ONT (%)	*E. coli* sim PacBio (%)	*E. coli* sim ONT (%)
No. of mapped reads	84.3	81	99.8	96
Avg identity GraphAligner	86.8	86.3	82.2	86.8
Avg identity SPAligner	86.7	86.3	82.2	86.6
	S. cerevisiae PacBio(%)	*S. cerevisiae* ONT (%)	*S. cerevisiae* sim PacBio (%)	*S. cerevisiae* sim ONT (%)
No. of mapped reads	51.8	60.1	98.7	84.4
Avg identity GraphAligner	86.9	82.5	82.4	82.4
Avg identity SPAligner	86.6	82.4	82.3	82.2

Supplementary Table S 3: Summary statistics on reads mapped by both SPAligner and GraphAligner. The table was generated by taking the intersection of sets of reads that were successfully mapped by SPAligner and GraphAligner. For each dataset and tool we identified percent of such reads and average identity of the resulting alignments.

9 Sequence-to-graph alignment implementation insights

This section provides a brief description of the GraphAligner tool, highlighting the differences between the design of SPAligner and GraphAligner. The description is based on a recently released preprint describing GraphAligner [14] and, since GraphAligner has been under active development some of the differences might not be relevant for the $v1.04$ version (current version $v1.10$) of the software that has been used in our benchmarks.

As well as SPAligner, GraphAligner starts with finding local similarities between the query sequence and individual label sequences. But while SPAligner uses BWA to detect longer anchor alignments, GraphAligner relies on the exact matches between a read and a node sequence (referred to as seeds). Earlier versions (including $v1.04$) used MUMmer4 [15] to identify the seeds, while the later versions (released after submission of this manuscript) moved to a novel strategy in which seeds are a certain subset of minimizer k-mers of the label sequences. In contrast to our approach, GraphAligner does not perform chaining of seed alignments and instead initiates alignment extension process from every seed (attempting to extend alignment to the entire read sequence from a single seed). Also while SPAligner filters all ambiguous nucleotide anchor alignments (ones with significant range query overlap), GraphAligner does not perform filtering of the ambiguously placed seeds. Instead the seeds are prioritized based on the number of their matches to the label sequences (extension is first initiated from the seeds having the fewer matches). As well as SPAligner GraphAligner uses a dynamic programming (DP) algorithm, which supports cyclic sequence graphs, to extend the alignments beyond the seeds/anchors.

GraphAligner features highly optimized implementation of optimal sequence-to-graph alignment (under unit costs model), extending earlier proposed graph alignment algorithm with bit-parallelism which is reported to achieve considerable practical speedup over previously suggested asymptotically optimal algorithms [16].

By default the seed matches that have been included into the alignment (while extending from a higher priority seed) are ignored. But this optimization can be disabled with an option to extend those seeds as well.

To avoid performance issues, the extension process uses several novel heuristics (for example to bound the time spent in tangled regions of the graph), some of which (s.a. the dynamic banding) are not unlike the ones used in SPAligner (see Supplemental Section "Alignment extension heuristics and thresholds").

Simple HMM model is used to determine whether the alignment extension should be terminated. Combined with the fact that the extension is performed from every seed, such boundary detection can be useful to account for regions of poor quality as well as structural differences between the query and the genome, represented by the assembly graph.

In the end a subset of identified alignment paths, corresponding to a non-overlapping query regions is greedily picked starting from the longest path. SPAligner uses similar approach to split-read alignment (see Supplement Section "Support for split-read alignments"), although for performance reasons we do this selection on chains of anchor alignments prior to the launch of dynamic programming extension procedure.

10 Dependence of alignment quality on read length

In order to understand how sequence-to-graph alignment quality changes with increase of read length, we decided to run SPAligner and GraphAligner on real *E. coli* ONT R9 dataset (See Supplementary Table S1) and simulated ONT data for *E. coli* and *C. elegans*. The model used for generating simulated data for both organisms was built from *E. coli* ONT R9 reads.

Reads from each dataset (real *E. coli*, simulated *E. coli*, simulated *C. elegans* were divided into 4 groups: "short" (length between 1 Kb and 10 Kb), "medium" (length between 10 Kb and 20 Kb), "long"(length between 20 Kb and 50 Kb), and "ultra-long" (length over 50 Kb). While for real *E. coli* dataset the number of "short", "medium" and "long" reads was 6000, 1200 and 800 respectively, the "ultra-long" group consisted of only 189 reads. Number of reads in each group from simulated dataset is 10000.

Metrics 'No.of mapped reads' and 'Average identity' described in Results Section were used for benchmarking.

For real *E. coli* dataset average identity value was around 87% for all groups of reads. Despite the fact that the fraction of aligned reads decreased from "short" to "ultra-long" group, for SPAligner results the difference between the groups was not large only 2% loss moving from "short" to "medium" (from 96% to 94%), less than 1% between "medium" and "long", and 6% loss moving from "long" to "ultra-long" one. In contrast fraction of mapped reads for GraphAligner decreased by almost 20% considering difference between "long" and "ultra-long" groups. Table S4 shows the summary results for running SPAligner and GraphAligner on reads of different length from real Ecoli dataset.

Results of SPAligner and GraphAligner on simulated data are more consistent in number of mapped reads on both *E. coli* and *C. elegans* simulated data. Average identity is around 87% as for original real dataset. The difference in the number of mapped reads only 5% between "short" and "ultra-long" groups for *E. coli* simulated data and 14% for more complex *C. elegans* dataset. Results summary can be found in Tables S5 and S6.

10.1 Real ONT reads alignment

Group	No. of reads	MR(%)	AI (%)	MR(%)	AI (%)
		SPAligner		GraphAligner	
1 – 10 Kb	5985	96.8	86.6	96.4	86.4
10 – 20 Kb	1268	95	87	91	86.7
20 – 50 Kb	774	94	87	83.2	87
> 50 Kb	189	88.9	86.9	64	87.1

Supplementary Table S 4: Number of mapped reads and average mapping identity of aligning *E. coli* ONT reads to short-read assembly graph (constructed by SPAdes with kmer size equal to 77) for queries of different lengths. SPAligner and GraphAligner results are provided for four groups groups of reads: "short" (1 – 10 Kb), "medium length" (10 – 20 Kb), "long" (20 – 50 Kb), and "ultra-long" (> 50 Kb). Based on its relatively poor performance (see Tables 1 and S1) we decided not to include the results of vg map into comparison.

10.2 Simulated ONT reads alignment

Group	MR(%)	AI (%)	MR(%)	AI (%)
	SPAligner		GraphAligner	
1 – 10 Kb	98.9	87	98.3	87.2
10 – 20 Kb	97.7	87.3	97.7	87.3
20 – 50 Kb	95.9	87.3	96	87.4
> 50 Kb	93.2	87.4	93.2	87.4

Supplementary Table S 5: Sequence-to-graph alignment of reads of different length for simulated *E. coli* data. Simulated reads were generated by Nanosim based on the model built on *E. coli* ONT R9 data described in Table S1. For each length we run Nanosim "simulator.py" command with corresponding "–min" and "–max" length parameters and seed 1234. While the parameters were set to produce 10000 reads for an unknown reason in all cases Nanosim generated 9747 reads.

Aligner	MR(%)	AI (%)	MR(%)	AI (%)
	SPAligner		GraphAligner	
1 – 10 Kb	98	86.9	97.9	87.2
10 – 20 Kb	94.9	87.2	95.7	87.4
20 – 50 Kb	91	87.2	92	87.4
> 50 Kb	83	87.2	85	87.4

Supplementary Table S 6: Sequence-to-graph alignment of reads of different length for simulated *C. elegans* data. Simulated reads were generated by Nanosim based on the model built on *E. coli* ONT R9 data described in Table S1. For each length we run Nanosim "simulator.py" command with corresponding "–min" and "–max" length parameters and seed 1234. While the parameters were set to produce 10000 reads for an unknown reason in all cases Nanosim generated 9747 reads.

References

[1] Sosic M, Sikic M. Edlib: a C/C++ library for fast, exact sequence alignment using edit distance;33(9):1394–1395. Available from: https://www.ncbi.nlm.nih.gov/pubmed/28453688.

[2] Amir A, Lewenstein M, Lewenstein N. Pattern Matching in Hypertext;35(1):82–99. Available from: https://linkinghub.elsevier.com/retrieve/pii/S0196677499910635.

[3] Antipov D, Korobeynikov A, McLean JS, Pevzner PA. hybridSPAdes: an algorithm for hybrid assembly of short and long reads;32(7):1009–1015. Available from: http://dx.doi.org/10.1093/bioinformatics/btv688.

[4] Navarro G. A guided tour to approximate string matching;33(1):31–88. Available from: http://portal.acm.org/citation.cfm?doid=375360.375365.

[5] Rautiainen M, Marschall T. Aligning sequences to general graphs in (+) time;Available from: http://biorxiv.org/lookup/doi/10.1101/216127.

[6] Nurk S, Bankevich A, Antipov D, Gurevich A, Korobeynikov A, Lapidus A, et al. Assembling Genomes and Mini-metagenomes from Highly Chimeric Reads. In: Deng M, Jiang R, Sun F, Zhang X, editors. Research in Computational Molecular Biology. vol. 7821. Springer Berlin Heidelberg;. p. 158–170. Available from: http://link.springer.com/10.1007/978-3-642-37195-0_13.

[7] Ono Y, Asai K, Hamada M. PBSIM: PacBio reads simulatortoward accurate genome assembly;29(1):119–121. Available from: http://dx.doi.org/10.1093/bioinformatics/bts649.

[8] Yang C, Chu J, Warren RL, Birol I. NanoSim: nanopore sequence read simulator based on statistical characterization;6(4):gix010–gix010. Available from: http://dx.doi.org/10.1093/gigascience/gix010.

[9] Huang W, Li L, Myers JR, Marth GT. ART: a next-generation sequencing read simulator;28(4):593–594. Available from: http://dx.doi.org/10.1093/bioinformatics/btr708.

[10] Tyson JR, O'Neil NJ, Jain M, Olsen HE, Hieter P, Snutch TP. MinION-based long-read sequencing and assembly extends the Caenorhabditis elegans reference genome;28(2):266–274. Available from: https://www.ncbi.nlm.nih.gov/pubmed/29273626.

[11] Salikhov K, Sacomoto G, Kucherov G. Using cascading Bloom filters to improve the memory usage for de Brujin graphs;9(1):2. Available from: http://almob.biomedcentral.com/articles/10.1186/1748-7188-9-2.

[12] Li D, Liu CM, Luo R, Sadakane K, Lam TW. MEGAHIT: an ultra-fast single-node solution for large and complex metagenomics assembly via succinct de Bruijn graph;31(10):1674–1676. Available from: http://dx.doi.org/10.1093/bioinformatics/btv033.

[13] Limasset A, Cazaux B, Rivals E, Peterlongo P. Read mapping on de Bruijn graphs;17(1). Available from: http://bmcbioinformatics.biomedcentral.com/articles/10.1186/s12859-016-1103-9.

[14] Rautiainen M, Marschall T. GraphAligner: Rapid and Versatile Sequence-to-Graph Alignment;Available from: http://biorxiv.org/lookup/doi/10.1101/810812.

[15] Marais G, Delcher AL, Phillippy AM, Coston R, Salzberg SL, Zimin A. MUMmer4: A fast and versatile genome alignment system;14(1):e1005944. Available from: https://dx.plos.org/10.1371/journal.pcbi.1005944.

[16] Rautiainen M, Mkinen V, Marschall T. Bit-parallel sequence-to-graph alignment;Available from: https://academic.oup.com/bioinformatics/advance-article/doi/10.1093/bioinformatics/btz162/5372677.
